# Supplementary material for: Current fossil fuel infrastructure does not yet commit us to 1.5 °C warming
Source: Nat Commun. 2019 Jan 15;10:101. doi: 10.1038/s41467-018-07999-w (PMC6333788; doi:10.1038/s41467-018-07999-w)
Supplement: Supplementary file 1 — Supplementary Information [file 41467_2018_7999_MOESM1_ESM.pdf]

Supplementary material to

Current infrastructure does not yet commit us to 1.5°C warming

Smith et al.

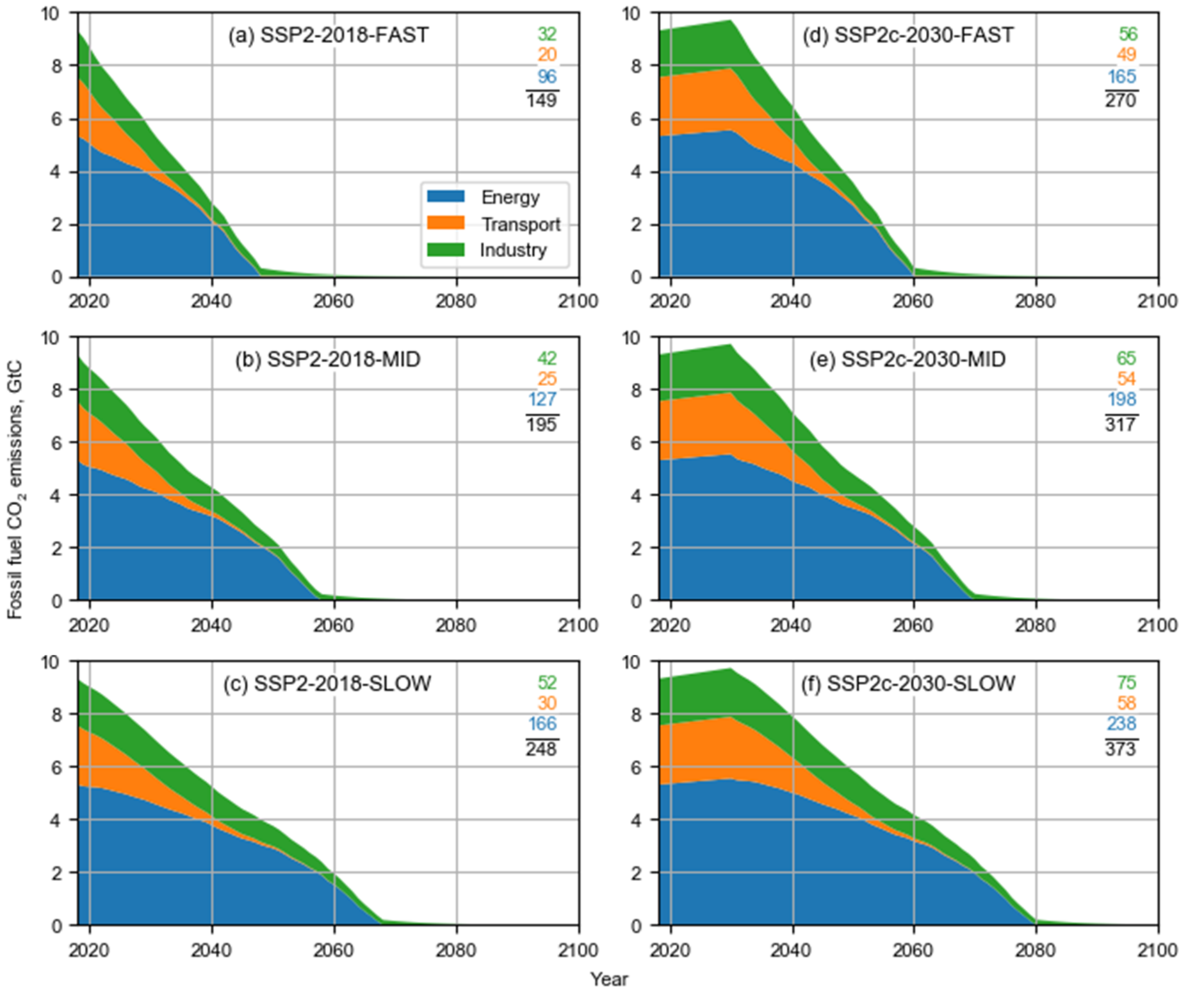

Supplementary Figure 1 | Fossil-fuel CO_2 emissions by sector in the 2018 and 2030 NDC-conditional infrastructure commitment scenarios. Numbers in the upper right of each panel give cumulative CO_2 emissions from 2018 in GtC from each sector plus the total in black (which may differ from the sum of components due to rounding). Phase-out scenarios are FAST, MID and SLOW based on a phase-out of energy sector emissions over 30, 40 or 50 years respectively. The “energy” sector includes contributions from minor sectors such as residential and services.

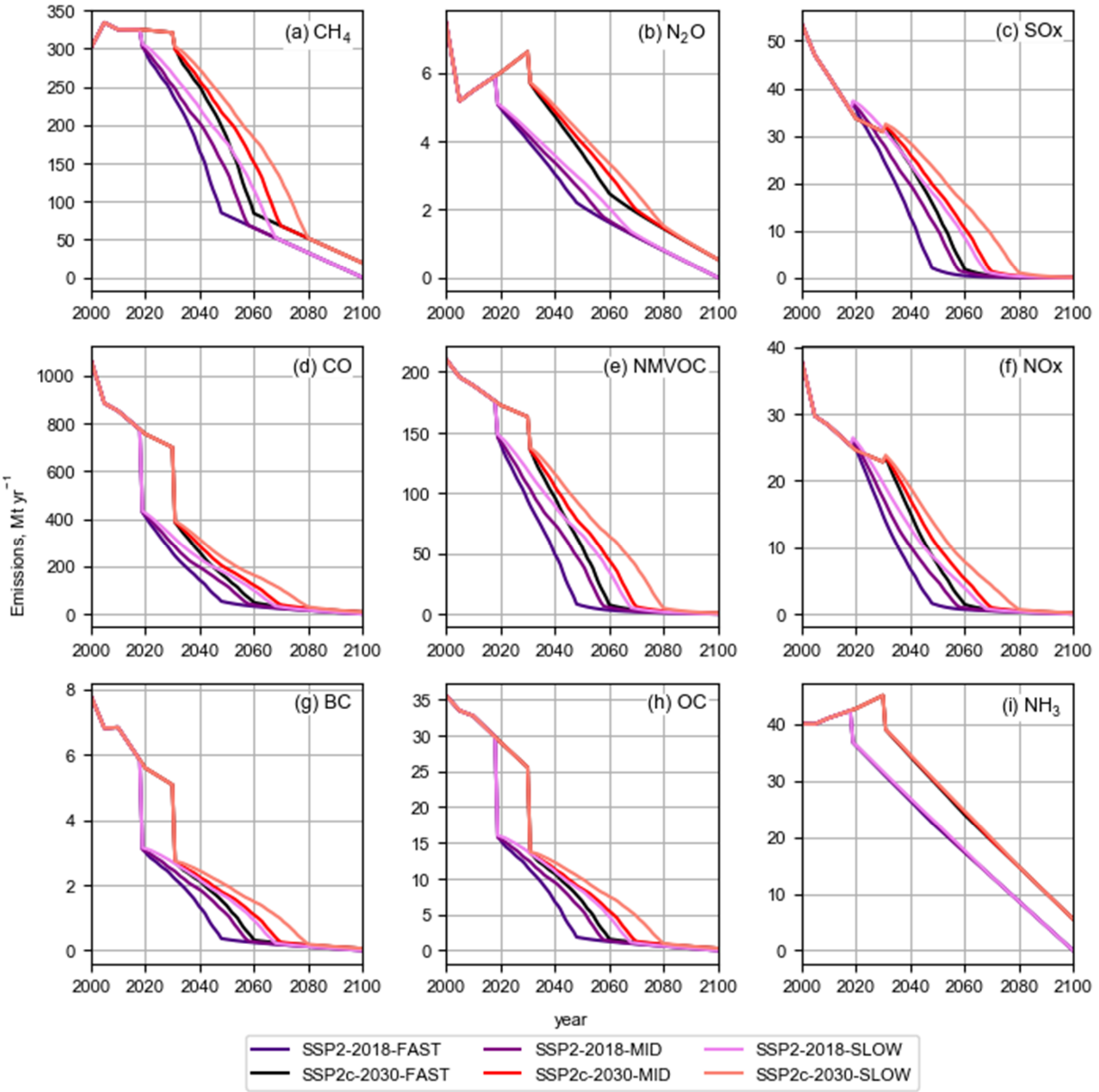

Supplementary Figure 2 | Emissions profiles of the major non-CO_2 greenhouse gases and short lived climate forcers in the 2018 and 2030 NDC-conditional infrastructure commitment scenarios.

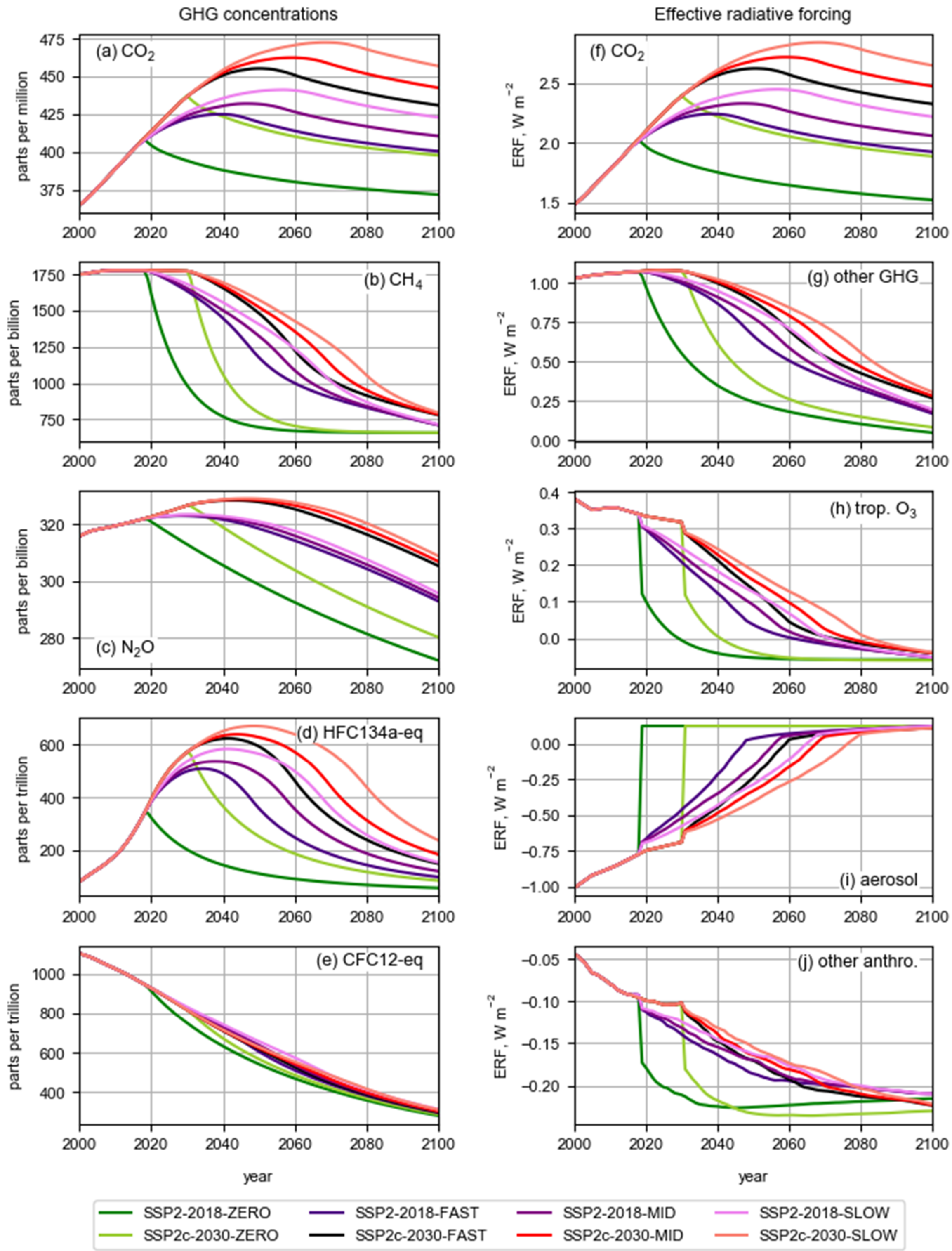

Supplementary Figure 3 | Atmospheric composition of greenhouse gases and effective radiative forcing diagnosed from the FaIR model for 2000 to 2100. Pathways are shown for the ZERO emissions commitments and the FAST, MID and SLOW phase-outs. Concentrations of (a) CO_2, (b) CH_4, (c) N_2O, (d) fluorinated gases as HFC134a-equivalent and (e) ozone depleting substances as CFC12-equivalent, and effective radiative forcing from (f) CO_2, (g) other well-mixed greenhouse gases, (h) tropospheric ozone, (i) aerosols and (j) other anthropogenic components (sum of land use change, stratospheric ozone, stratospheric water vapour from methane oxidation, aviation contrails and black

carbon on snow). In (a) and (f)-(j), the median values from the distributions of CO_2 concentrations and effective radiative forcing of all components are shown. In the ZERO emissions commitments, aerosol ERF is positive and tropospheric ozone ERF is negative in 2100, because zero ERF is defined to be 1750 levels, and ozone and aerosol precursor emissions were small but non-zero in 1750 (Skeie et al., 2011).

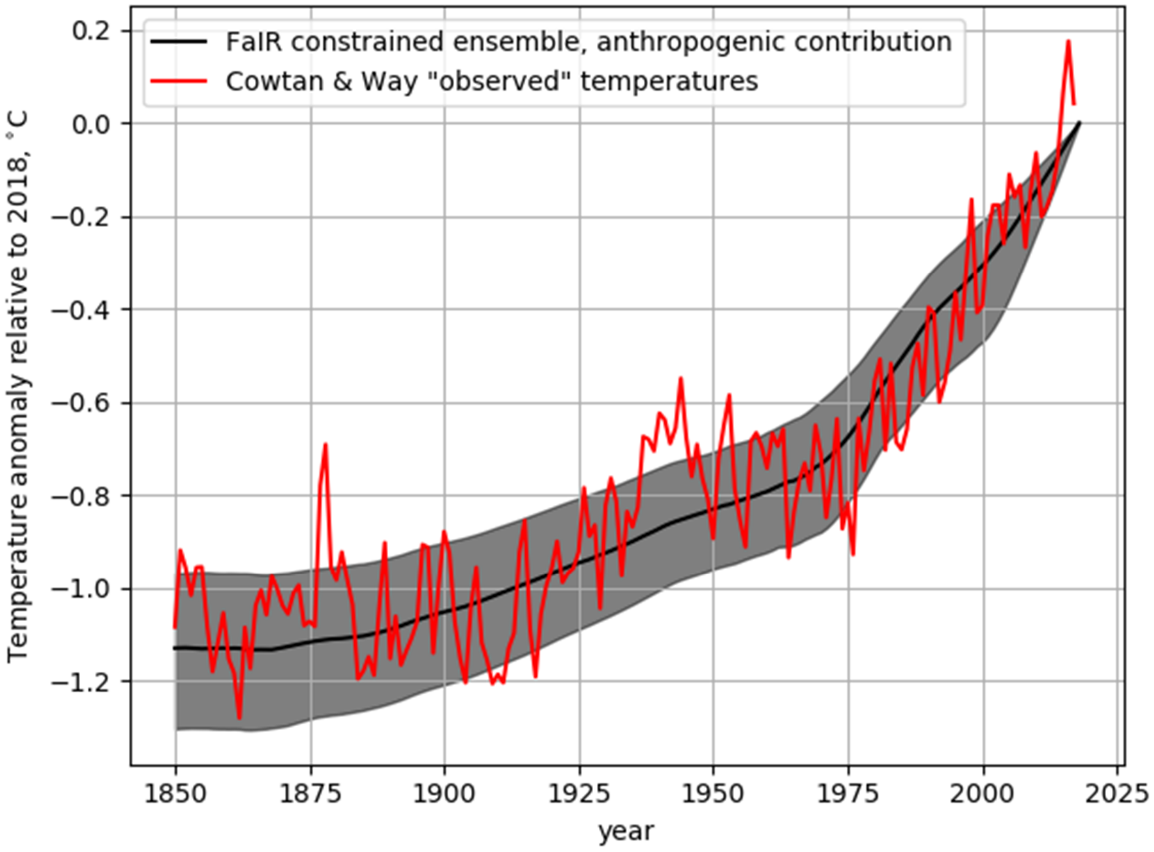

Supplementary Figure 4 | Correspondence of modelled anthropogenic-only temperatures from FaIR (1850-2018) to observations from Cowtan & Way for 1850-2017. Temperatures are shown with a 2018 baseline. The shaded region shows the 5-95% range of the constrained FaIR ensemble.

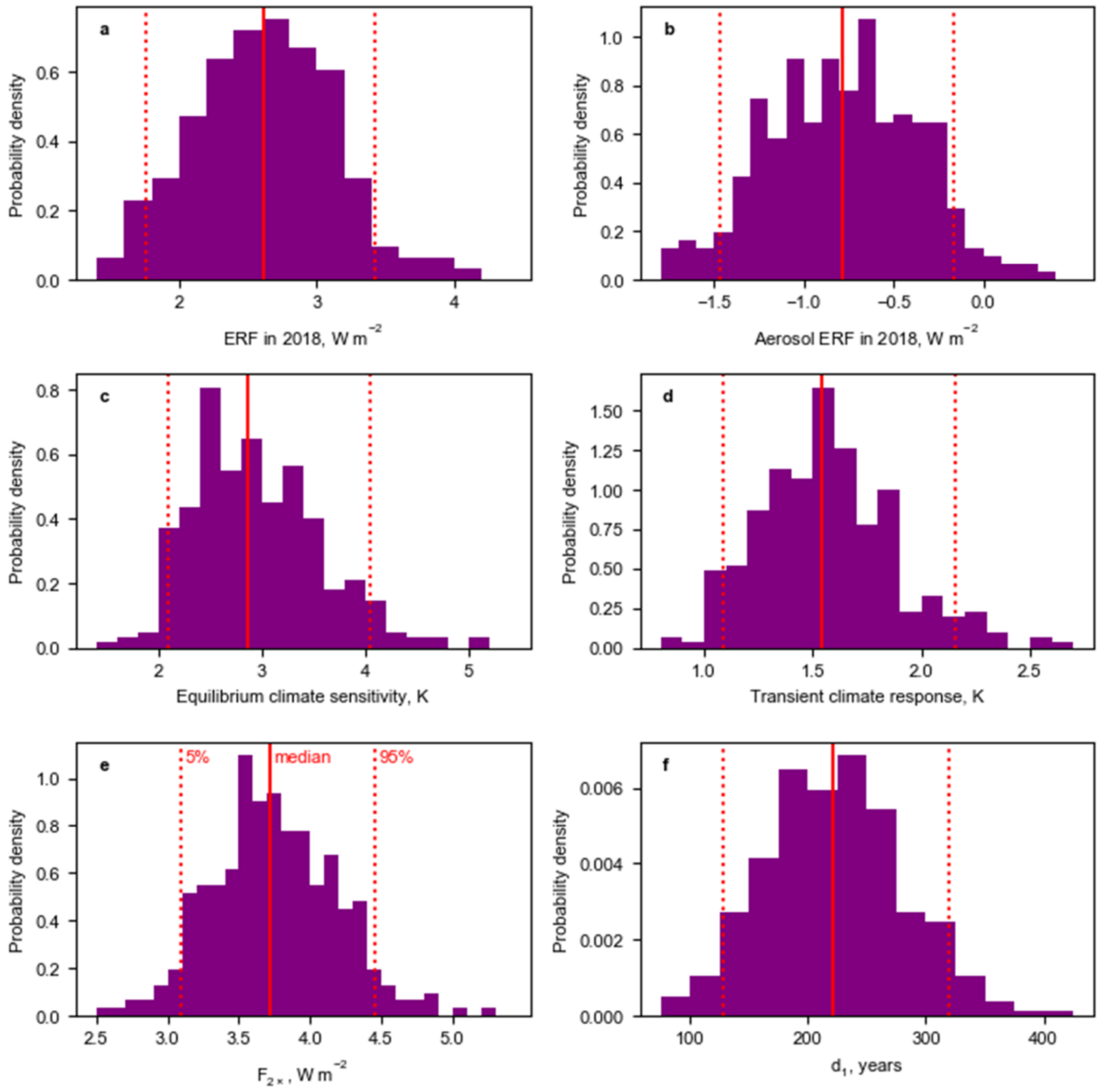

Supplementary Figure 5 | Posterior distributions of parameters in the FaIR model. (a) Total effective radiative forcing in 2018; (b) Aerosol ERF in 2018; (c) Equilibrium climate sensitivity; (d) Transient climate response; (e) ERF from a doubling of CO_2; (f) time constant of the deep ocean thermal response.

Supplementary Figure 6 commentary

We repeat the experiments of Matthews and Zickfeld (2012) (who used the UVic2.9 model), for 21st century temperature change from zeroing emissions of CO_2, zeroing all GHG emissions, and zeroing CO_2 and aerosol emissions from 2018 onwards, alongside SSP2-2018-ZERO (Supplementary Fig. 6). We show effective radiative forcing (Supplementary Figs. 6a and 6b) and temperature change from 2018 (Supplementary Figs. 6c and 6d). Constant 2018 forcing from non-zeroed components is assumed. Similar qualitative behaviour between our study and Matthews and Zickfeld (2012) is observed for an abrupt zeroing of CO_2 emissions (slight warming in the ensemble median to 2100; red curve), zeroing all GHG emissions (moderate cooling; yellow curve), and zeroing CO_2 and aerosol emissions (substantial warming; blue curve), but with a smaller difference between the 2100 and present-day temperatures than in Matthews and Zickfeld (2012). These differences are due to a higher temperature sensitivity of carbon sinks in UVic2.9 compared to FaIR (Millar et al., 2017), which act to amplify the warming or cooling.

In contrast to Matthews and Zickfeld (2012) we find an end-of-century net cooling, rather than a net warming, for a zero emissions commitment along with a smaller near-term and peak warming (green curve in Supplementary Fig. 6). Alongside the lower sensitivity of carbon sinks to temperature in FaIR, this is also due to the lower range of present-day aerosol forcing (5 to 95% range of -0.2 to -1.4 W m^{-2}; Supplementary Fig. 5b) from our constrained projections, compared to the -1.0 to -1.5 W m^{-2} range of Matthews and Zickfeld (2012).

In addition we show the temperature response from a simple linear phase-out of CO_2 emissions to a time of net zero emissions with constant 2018 non-CO_2 forcing, which reaches 1.5°C at the point of zero CO_2 emissions. We find this empirically to be 48 years. Beyond the year of zero CO_2 emissions (2066), temperatures continue to rise slightly (red dotted curve, Supplementary Fig. 6), unlike in SSP2-2018-MID, in which temperatures slowly decline from their mid-century peak (purple curve, main Fig. 1a). This highlights the importance of non-CO_2 forcing on long-term temperature projections.

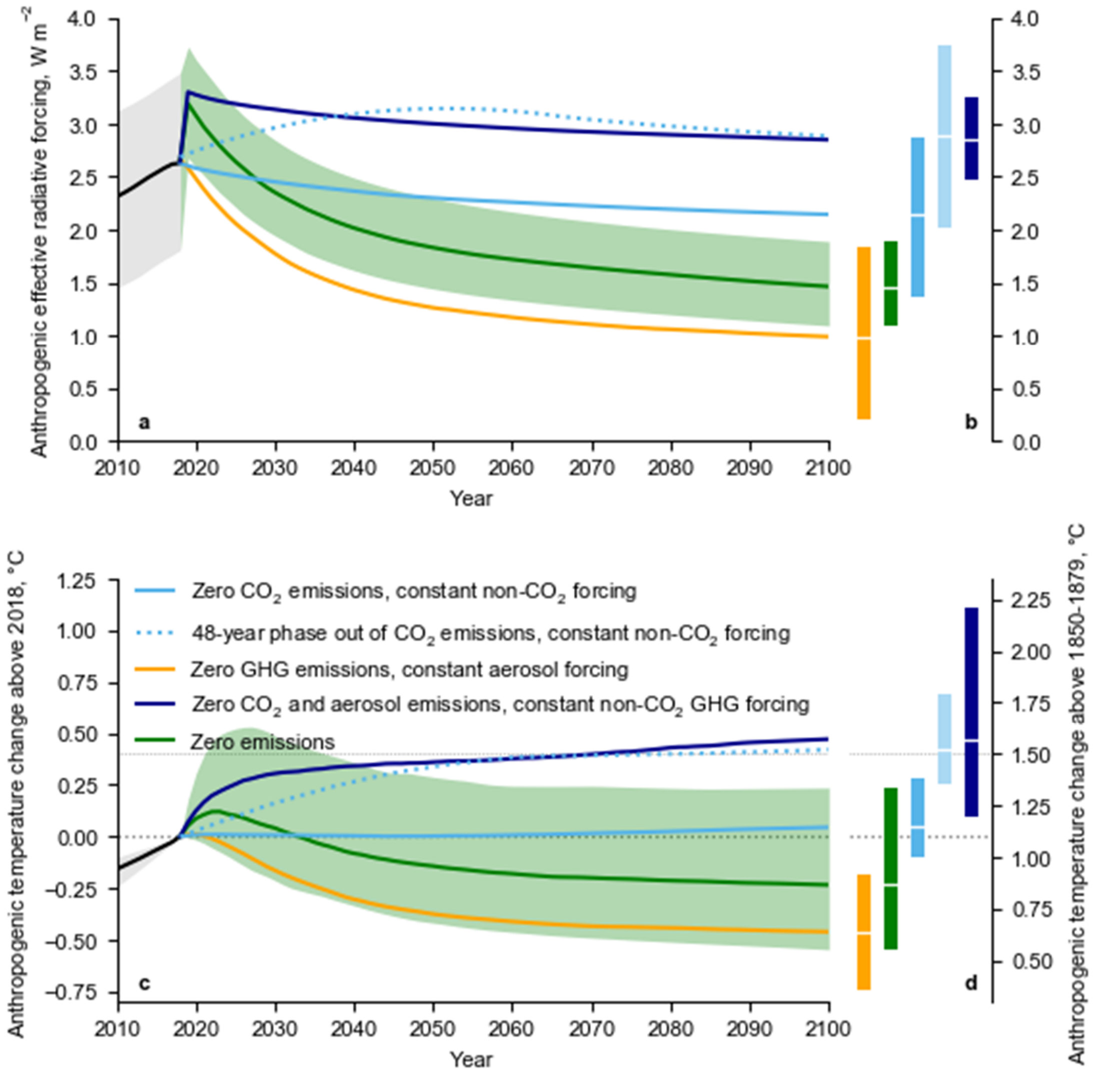

Supplementary Figure 6 | Effects of setting different categories of emissions to zero. **a,** Anthropogenic effective radiative forcing over 2010 to 2100; lines represent median of 1000-member constrained ensemble, shaded area is 5th-95th percentiles of distribution of SSP2-2018-ZERO. **b,** 5 to 95% ensemble ranges (coloured bars) and median (white lines) of anthropogenic ERF definition in 2100. **c,** and **d,** as panel (a) and (b), but for anthropogenic component of temperature change.

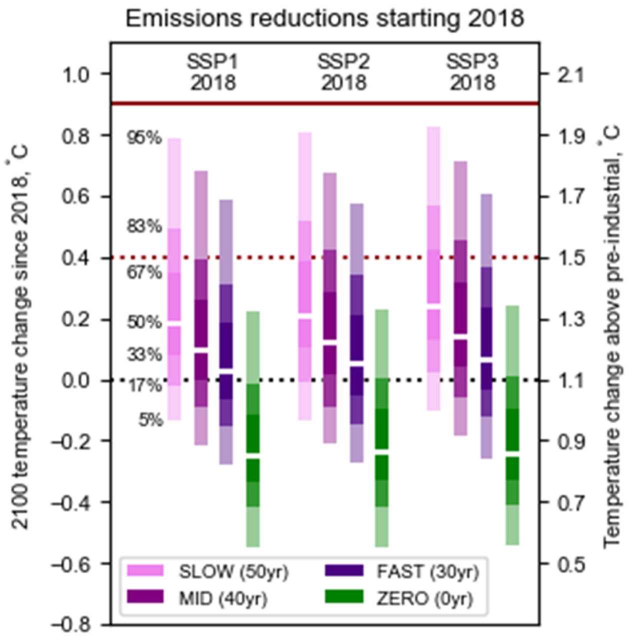

Supplementary Figure 7 | 2100 century temperature anomaly compared to 2018 for different rates of fossil fuel phase-out (FAST, MID and SLOW) plus an abrupt cessation of all emissions (ZERO) from 2018. MID scenarios assume a phase-out of fossil fuel infrastructure based on historical generator lifetimes (Davis and Socolow, 2014). FAST and SLOW cases vary these lifetimes by subtracting and adding 10 years, respectively. Shown are results for emissions under SSP1, SSP2, and SSP3 until 2018.

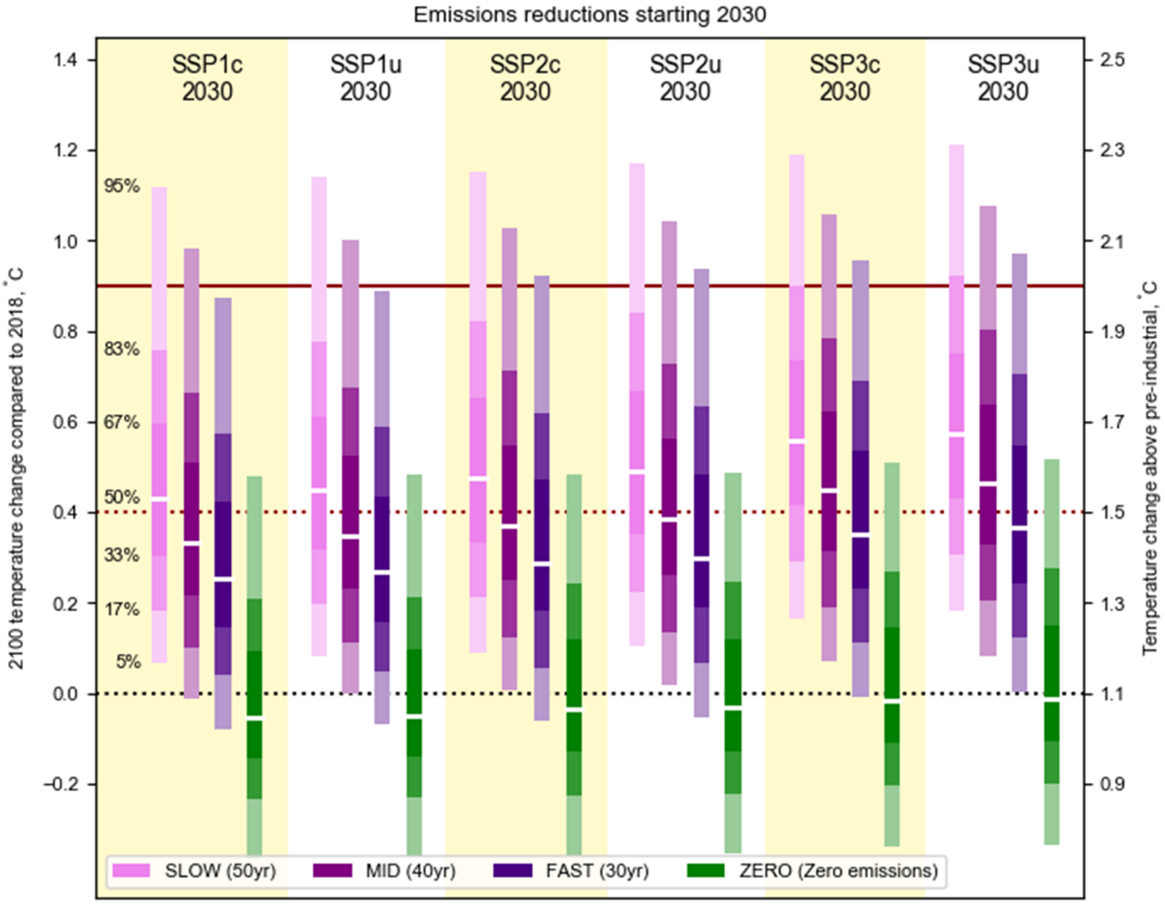

Supplementary Figure 8 | 2100 temperature anomaly compared to 2018 for different rates of fossil fuel phase-out (MAX, MID and MIN) plus an abrupt cessation of all emissions (ZERO) from 2030. MID scenarios assume a phase-out of fossil fuel infrastructure based on historical generator lifetimes (Davis and Socolow, 2014). MAX and MIN cases vary these lifetimes by subtracting and adding 10 years, respectively. Shown are results from SSP1, SSP2 and SSP3 with for conditional and unconditional NDCs based on socio-economic developments under SSP1, SSP2 and SSP3 (Rogelj et al., 2017).

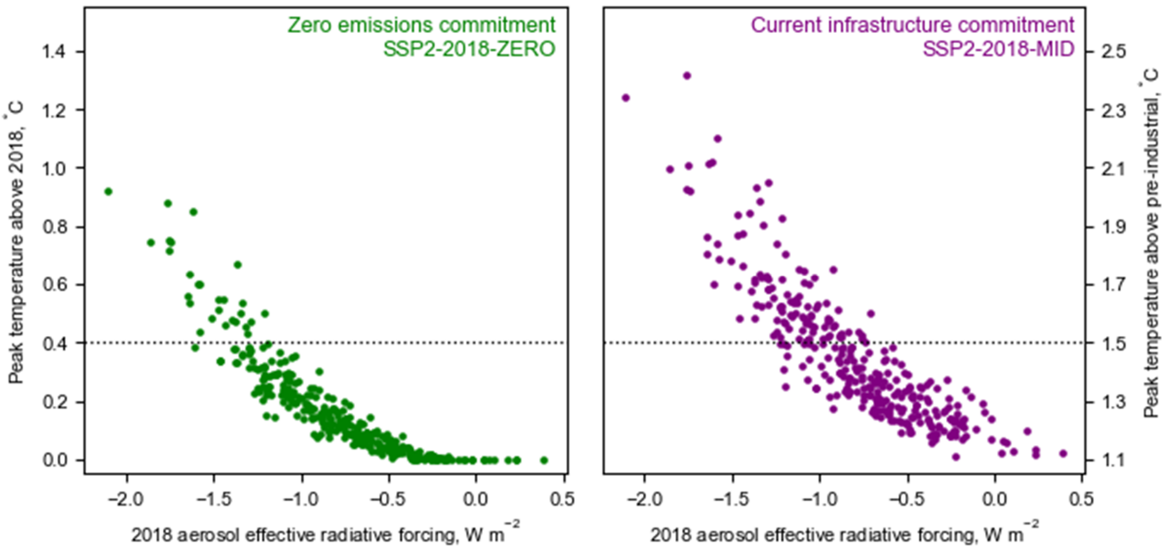

Supplementary Figure 9 | Relationship between present-day aerosol effective radiative forcing and peak temperature. Left panel shows the peak temperature above present day for the zero emissions commitment SSP2-2018-ZERO and right panel shows peak temperature for the infrastructure commitment SSP2-2018-MID.

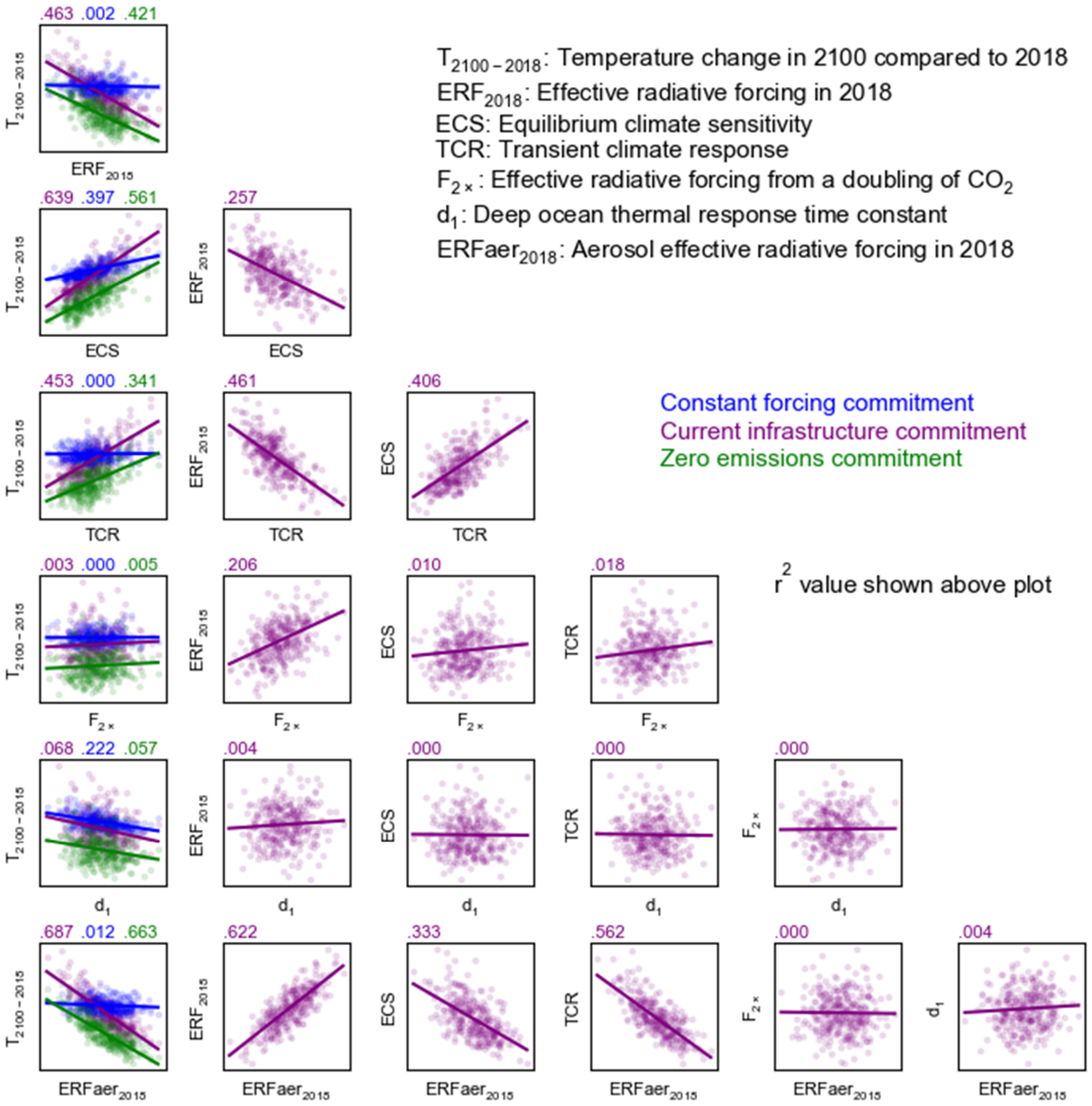

Supplementary Figure 10 | Correlations between model parameters and year 2100 temperature. First column shows the correlations between each of ERF; ECS; TCR; $F_{2\times}$; deep ocean thermal time constant (d_1); and aerosol ERF, and year-2100 temperature. Blue points and regression slopes are for SSP2-2018-CONST (blue), SSP2-2018-MID (purple) and SSP2-2018-ZERO (green) commitments. The matrix plot shows correlations between individual parameters, which are independent of commitment as they are constrained over the historical period. Numbers above each panel show the correlation coefficients.

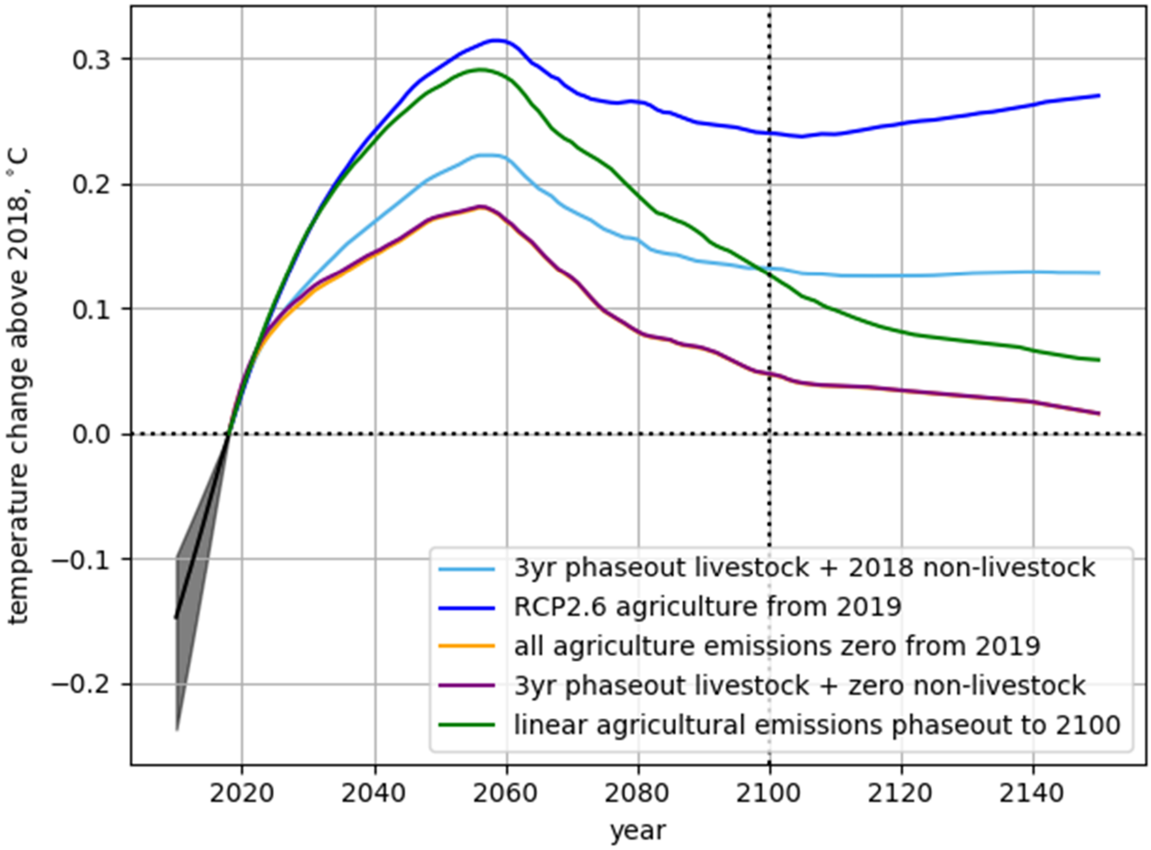

Supplementary Figure 11 | Sensitivity of future temperature change to different agricultural emissions scenarios. The underlying fossil-fuel phase-out scenario is SSP2-2018-MID. The "all agriculture emissions zero" curve lies behind the "3yr phaseout livestock + zero non-livestock" curve. For all phase-out scenarios in the analysis we use the green curve, representing a 3-year phase-out of livestock related emissions and an 82-year phase-out of all non-livestock agricultural emissions. For the zero emissions scenarios we also set the agricultural emissions immediately to zero (orange curve, noting that the underlying profile in this figure is not a zero-emissions commitment).

Phase-out scenario	Annual rate of decarbonisation (Gt C yr^{-1})
SSP1-2018-FAST	0.29
SSP1c-2030-FAST	0.30
SSP1u-2030-FAST	0.31
SSP2-2018-FAST	0.30
SSP2c-2030-FAST	0.31
SSP2u-2030-FAST	0.32
SSP3-2018-FAST	0.33
SSP3c-2030-FAST	0.36
SSP3u-2030-FAST	0.37
SSP1-2018-MID	0.22
SSP1c-2030-MID	0.23
SSP1u-2030-MID	0.24
SSP2-2018-MID	0.23
SSP2c-2030-MID	0.24
SSP2u-2030-MID	0.25
SSP3-2018-MID	0.25
SSP3c-2030-MID	0.28
SSP3u-2030-MID	0.29
SSP1-2018-SLOW	0.18
SSP1c-2030-SLOW	0.18
SSP1u-2030-SLOW	0.19
SSP2-2018-SLOW	0.18
SSP2c-2030-SLOW	0.19
SSP2u-2030-SLOW	0.20
SSP3-2018-SLOW	0.20
SSP3c-2030-SLOW	0.22
SSP3u-2030-SLOW	0.23

Supplementary Table 1 | Approximate rate of decarbonisation in each phase-out scenario. Rates of decarbonisation calculated as a linear trend from the first year of commitment to year 30 (FAST), 40 (MID) or 50 (SLOW).

**Parameter**	**5 to 95 percentile range (percent of best estimate)**	**Distribution**
ERF from CO_2 (radiative forcing from a doubling of CO_2)	±20% (3.71 ± 0.74 W m^{-2})	Gaussian
ERF from CH_4	±28%	Gaussian
ERF from N_2O	±20%	Gaussian
ERF from minor greenhouse gases	±20%	Gaussian
ERF from tropospheric O_3	±50%	Gaussian
ERF from stratospheric O_3	±200%	Gaussian
ERF from stratospheric water vapour from CH_4 oxidation	±72%	Gaussian
ERF from contrails	-66% to 191%	Lognormal
ERF from aerosols	-89% to 111%	Two half-Gaussians for regions above and below median
ERF from black carbon on snow	-56% to 128%	Lognormal
ERF from land use change	167%	Gaussian
Equilibrium climate sensitivity (ECS)	2.0 to 4.9°C (median 3.1°C)	Joint lognormal with TCR (correlation coefficient 0.81), informed by distribution of CMIP5 models
Transient climate response (TCR)	1.0 to 3.0°C (median 1.7°C)	
Pre-industrial 100-year time integrated airborne fraction of CO_2 ($iIRF_{100}$)	29.0 to 37.6 yr (median 33.3 yr)	Gaussian
Increase in $iIRF_{100}$ as a function of cumulative carbon emission	0.0165 to 0.0215 yr GtC^{-1} (median 0.019 yr GtC^{-1})	Gaussian
Increase in $iIRF_{100}$ as a function of Industrial Era temperature change	3.62 to 4.70 yr °C^{-1} (4.16 yr °C^{-1})	Gaussian
Time constant of temperature response of land surface and ocean mixed layer	2.7 to 5.5 yr (4.1 yr)	Gaussian, truncated to ±3σ
Time constant of temperature response of deep ocean	105.9 to 313.1 yr (209.5 yr)	Gaussian, truncated to ±3σ

Supplementary Table 2 | Variation of input parameters to the FaIR climate model.

Sector	CH_4	N_2O	SO_2	CO	VOC	NOx	BC	OC	NH_3
Energy	33%	3%	51%	3%	25%	29%	16%	16%	2%
Industry		12%	31%	12%	18%	11%	6%	6%	1%
Road transport		2%	1%	15%	13%	21%	1%	1%	1%
Aircraft + shipping		1%	9%	1%	1%	18%	2%	2%	
Livestock	31%	3%					1%	1%	21%
Other agriculture	11%	53%		8%	3%	4%	7%	7%	62%
Biomass	5%	13%	2%	44%	15%	12%	47%	47%	12%
Other sectors	20%	13%	7%	18%	25%	5%	21%	21%	

Supplementary Table 3 | Proportion of non-CO_2 emissions attributable to each sector used in the first year of the phase-out scenarios. Emissions data is from 2008, from the EDGAR database (European Commission Joint Research Center and Netherlands Environmental Assessment Agency, 2014). Blank cells correspond to 0%. Columns may not sum to 100% due to rounding.

Parameter	Minimum and maximum range
2017 CO_2 ERF (ERF from a doubling of CO_2)	1.42 to 2.84 (2.6 to 5.2) W m^{-2}
2017 CH_4 ERF	0.29 to 0.88 W m^{-2}
2017 N_2O ERF	0.11 to 0.21 W m^{-2}
2017 ERF from other well-mixed greenhouse gases	0.23 to 0.44 W m^{-2}
2017 ERF from tropospheric O_3	0.01 to 0.45 W m^{-2}
2017 ERF from stratospheric O_3	-0.14 to +0.08 W m^{-2}
2017 ERF from stratospheric water vapour from CH_4 oxidation	-0.03 to 0.20 W m^{-2}
2017 ERF from contrails	0.00 to 0.35 (0.05) W m^{-2}
2017 ERF from aerosols	-2.24 to +0.40 W m^{-2}
2017 ERF from black carbon on snow	0.00 to 0.14 W m^{-2}
2017 ERF from land use	-0.43 to +0.06 W m^{-2}
Equilibrium climate sensitivity (ECS)	1.5 to 5.1 °C
Transient climate response (TCR)	0.8 to 2.7 °C
Pre-industrial 100-year time integrated airborne fraction of CO_2 ($iIRF_{100}$)	26 to 41 yr
Sensitivity of $iIRF_{100}$ to cumulative carbon emissions	0.015 to 0.024 yr GtC^{-1}
Sensitivity of $iIRF_{100}$ to temperature change	2.9 to 5.1 yr $°C^{-1}$
Time constant of temperature response of land surface and ocean mixed layer	1.4 to 7.0 yr
Time constant of temperature response of deep ocean	54 to 402 yr

Supplementary Table 4 | Lower and upper bounds of each parameter in the variance-based sensitivity analysis. The lower and upper bounds are chosen that produces at least one ensemble member within the Cowtan and Way (2014) observed warming trend of 0.95 ± 0.17°C from 1880 to 2016 in a full historical simulation including natural forcing. Rows of the table are colour-coded in correspondence with the bars in Figure 5 (main manuscript). All 18 parameters are allowed to vary independently, with parameters that contribute only a small fraction of overall variance aggregated.

References

Cowtan, K. and Way, R.G. 2014. Coverage bias in the HadCRUT4 temperature series and its impact on recent temperature trends. *Q. J. Roy. Meteor. Soc.* **140**, pp.1935-1944.

Davis, S.J. and Socolow, R.H. 2014. Commitment accounting of CO_2 emissions. *Environmental Research Letters.* **9**, p084018.

European Commission Joint Research Center and Netherlands Environmental Assessment Agency. 2014. Emission Database for Global Atmospheric Research (EDGAR), release EDGARv4.2 FT2012. [Online]. Available from: http://edgar.jrc.ec.europa.eu/overview.php?v=42FT2010

Matthews, H.D. and Zickfeld, K. 2012. Climate response to zeroed emissions of greenhouse gases and aerosols. *Nat. Clim. Change.* **2**, pp.338-341.

Millar, R.J., Nicholls, Z.R., Friedlingstein, P. and Allen, M.R. 2017. A modified impulse-response representation of the global near-surface air temperature and atmospheric concentration response to carbon dioxide emissions. *Atmos. Chem. Phys.* **2017**, pp.7213-7228.

Rogelj, J., Fricko, O., Meinshausen, M., Krey, V., Zilliacus, J.J.J. and Riahi, K. 2017. Understanding the origin of Paris Agreement emission uncertainties. *Nature Communications.* **8**, p15748.

Skeie, R.B., Berntsen, T.K., Myhre, G., Tanaka, K., Kvalevåg, M.M. and Hoyle, C.R. 2011. Anthropogenic radiative forcing time series from pre-industrial times until 2010. *Atmos. Chem. Phys.* **11**(22), pp.11827-11857.
